# Cloning and functional analysis of the *BrCUC2* gene in *Brassica rapa* L

**DOI:** 10.3389/fpls.2023.1274567

**Published:** 2023-10-30

**Authors:** Xiaolei Tao, Yuhong Zhao, Li Ma, Junyan Wu, Rui Zeng, JinTang Jiao, Rong Li, Weiming Ma, Yintao Lian, Wangtian Wang, Yuanyuan Pu, Gang Yang, Lijun Liu, Xuecai Li, Wancang Sun

**Affiliations:** ^1^ State Key Laboratory of Aridland Crop Science, College of Agronomy, Gansu Agricultural University, Lanzhou, China; ^2^ Gansu Key Laboratory of Crop Genetic Improvement and Germplasm Innovation, Gansu Agricultural University, Lanzhou, China; ^3^ Gansu Yasheng Agricultural Research Institute Co. Ltd, Crop Office, Lanzhou, China

**Keywords:** Brassica rapa, BrCUC2, functional analysis, low-temperature stress, transgenic Arabidopsis thaliana

## Abstract

The *CUP-SHAPED COTYLEDON2 (CUC2)* gene plays an important role in the formation of apical meristem and organ edges in plants. The apical meristematic tissue of *Brassica rapa* (*B. rapa*) is associated with cold resistance, however, the role of the *CUC2* gene in cold resistance of *B.rapa* is unclear. In this study, we used bioinformatics software to analyze the structure of *BrCUC2* gene, real-time fluorescence quantitative PCR to detect the expression level of *BrCUC2*, constructed transgenic *Arabidopsis thaliana* by the flower dipping method and subcellular localization for functional validation. The results showed that, we isolated a 1104 bp open reading frame of *BrCUC2* from the winter *B. rapa* cultivar ‘Longyou 7’. The BrCUC2 contains a highly conserved domain belonging to the NAM superfamily. Its homologus *CUC* genes contain similar conserved motifs and are closely related to *Brassica oleracea* (*B.oleracea*), and the N-terminal of amino acid sequence contains NAC domain. BrCUC2 protein was localized in the nucleus and self-activation tests showed that pGBKT7-BrCUC2 had self-activation. Tissue-specific expression analysis and promoter β-Glucuronidase (GUS) activity showed that *BrCUC2* had high expression levels in *B. rapa* growth points and *A. thaliana* leaf edges, stems and growth points. After low-temperature stress, *BrCUC2* showed greater expression in ‘Longyou 7,’ which presents strong cold resistance and concave growth points, than in ‘Longyou 99,’ which presents weak cold resistance and protruding growth points. *BrCUC2* promoter contains multiple elements related to stress responses. *BrCUC2* overexpression revealed that the phenotype did not differ from that of the wild type during the seedling stage but showed weak growth and a dwarf phenotype during the flowering and mature stages. After low-temperature treatment, the physiological indexes and survival rate of *BrCUC2*-overexpression lines of *Arabidopsis thaliana* (*A. thaliana*) were better than those of the wild type within 12 h, although differences were not observed after 24 h. These results showed that *BrCUC2* improved the low-temperature tolerance of transgenic *A. thaliana* within a short time. It can provide a foundation for the study of cold resistance in winter *B. rapa*.

## Introduction

Rapeseed production is important for the sustainable supply of edible oil in China (Li et al., 2022). In northern China, the temperature can reach -32°C in winter, drought occurs in spring, and ecological conditions are harsh (Zhou et al., 2014). Wind erosion and farmland degradation are severe, and desertified areas are expanding. The successful northward migration of winter rapeseed, which represents the only winter oil seed crop, has provided economic benefits in the north (Ma et al., 2019b). Moreover, the plant has been utilized as a winter cover crop, which can effectively increase vegetation coverage in winter and spring, improve the multiple cropping index and land utilization rate, reduce soil wind erosion, increase organic matter content, protect farmlands, promote balanced development between local economic production, and achieve ecological and environmental protection(Guo et al., 2023).

As the main production region for winter rapeseed, the planting area of northern China accounts for approximately 90% of the total rapeseed area. *Brassica rapa* (*B. rapa*) is mainly distributed on the western Loess Plateau (Wu et al., 2022). Before overwintering, the aboveground parts of strong cold-resistant varieties grow slowly, and the leaves grow prostrate and enter the withered leaf stage earlier (Ma et al., 2019a). During the overwintering period, the growth point of winter rapeseed varieties with strong cold resistance sinks, and the growth occurs belowground. The central leaves cover the surface, such that the growth point can maintain a specific moisture content and remain in the soil layer at a relatively stable temperature. This can effectively prevent damage to rapeseed when the temperature changes dramatically and allow the plant to safely overwinter. Sun et al (Sun et al., 2011a) found that winter rapeseed varieties with different cold resistance have great differences in the growth points, and the strong cold resistance *B. rapa* varieties that can overwinter in northern regions such as Hexi Corridor in Gansu Province have the characteristics of concave growth points and creeping growth. (Niu et al., 2021). Therefore, an unknown regulatory mechanism may occur between the morphological characteristics of winter rapeseed with concave and raised growth points and their cold resistance.

The NAC family transcription factor *CUP-SHAPED COTYLEDON2* (*CUC2*) regulates the formation of various tissues and organs during plant growth and development (Hu et al., 2015), and plays a particularly important role in the formation of the apical meristem and maintenance of organ primordium edges (Aida et al., 1997; Olsen et al., 2005). In 1997, the *CUC2* gene was first isolated and identified in *Arabidopsis thaliana* (*A. thaliana*) (Niu et al., 2021), and its mutation caused developmental defects in the apical meristem (Aida et al., 1999; Maugarny et al., 2016). The *CUC2* gene can promote the aggregation of auxins in the organ primordium by regulating *PIN-FORMED 1* (*PIN1*), thus participating in the regulation of early ovule primordium formation (Kwon et al., 2006). In addition, *CUC2* can indirectly regulate the synthesis of *LATERAL SUPPRESSOR* (*LAS*) genes for lateral branch development (Reinhardt et al., 2003).

*CUC2* is a negative regulator of plant growth that antagonizes cytokinins and auxins (Motte et al., 2011). It slows plant growth by inhibiting the division of plant cells and promoting the initiation of apical meristem development under the regulation of auxin. *CUC2* is gradually expressed and leads to changes in the plant growth stage, beginning in the spherical embryo stage and proceeding to the tip of the embryo during seed germination and the edge of the cotyledon after cotyledon formation (Nikovics et al., 2006). As the upstream gene of the apical meristem-forming factor Shoot meristemless (*STM*) (Balkunde et al., 2017), the *CUC2* gene is regulated by the activity of the *STM* gene (Spinelli et al., 2011) during the growth and development of the apical meristem of *A. thaliana* plants and restricted to the margins of the cotyledon and apical meristem. Apical meristem formation is regulated by regulating the expression of *STM* (Balkunde et al., 2017). Studies on *CUC2* promoters of *Betula platyphylla* (*B. platyphylla*) found that they contain many cis-acting elements associated with tissue-specific expression, hormone synthesis, environmental stress response, and transcription factors and are highly expressed in the apical meristem, leaf margins, and flower tissue organs (Aida et al., 1999).

The regulatory role of the *CUC2* gene in *A. thaliana* and *B. platyphylla* meristems has been studied. *CUC2* is a negative regulator of plant growth that plays a role in the apical meristem and organ edge formation, leaf edge morphogenesis, and growth and development (Aida et al., 1997). However, its role in the growth point of winter *B. rapa* remains unclear.

In this study, we cloned the *BrCUC2* gene from the growing point of winter *B. rapa* and analyzed its bioinformatics and tissue expression. The promoter sequence 2000 bp upstream of *BrCUC2* was cloned using Gateway technology. Online software was used to predict the cis-acting regulatory element of the promoter, and a promoter expression vector was constructed and transformed into *A. thaliana.* Transgenic plants were then identified by β-Glucuronidase (GUS) histochemical staining. This study reveals the role of *BrCUC2* at the growth point of winter *B. rapa* and lays a foundation for further studies on the transcriptional regulation of *BrCUC2*.

## Materials and methods

### Plant materials and growth conditions

The experimental *B. rapa* materials included the ‘Longyou 7’ (strong cold resistance) and ‘Longyou 99’ (weak cold resistance) varieties. Rapeseed seeds with excellent vitality were selected, treated with 10% H_2_O_2_ for 30 min, rinsed with sterile water 2–3 times, and germinated in Petri dishes containing wet filter paper under light conditions for 14 h at 30°C and dark conditions for 10 h at 28°C. After germination, the seeds were sown in pots and cultured in an artificial incubator (14 h light at 25°C, 10 h dark at 20°C) until the seven–leaves stage. Rapeseed seedlings were placed in incubators at 4, 0, and -4°C for the low-temperature treatment and 25°C (room temperature) as the control. After 72 h of treatment, the roots, stems, leaves, and growth cones of rapeseed were collected, immediately frozen in liquid nitrogen, and stored at -80°C for real-time fluorescence quantitative expression analysis of extracted RNA. Three biological replicates were performed for each treatment.

*Nicotiana benthamiana* (*N.benthamiana*) seeds were vernalized at 4°C for three days and seeded in a sterilized soil matrix. A subcellular localization test was performed during the 4th week of growth. The growth chamber conditions were as follows: 16 h light, 8 h darkness, 25°C, and 65% relative humidity.

### Cloning of the *BrCUC2* gene

The basic local allignment search tool (BLAST) alignment method of the national center for biotechnology information (NCBI) was applied to the *BrCUC2* gene coding region sequence reported in the GenBank database, and a coding sequence with 100% homology to *B. rapa* was obtained. Primer Premier 5.0 software (Premier Biosoft International, Palo Alto, CA, USA) was used to design the gene cloning primers *CUC2*-A: 5′-ATGGACATTCCGTACTACCACTAT-3′ and *CUC2*-S: 5′-TAGTAATTCCATACGCAATCAAGT-3′. The cDNA at the ‘Longyou 7’ growing point at room temperature was used as a template for *in vitro* amplification by polymerase chain reaction (PCR). The amplification process was as follows: pre-denaturation at 94°C for 5 min, followed by 35 cycles of denaturation at 94°C for 30 s, annealing at 58.4°C for 30 s, and extension at 72.0°C for 60 s; terminal extension at 72°C for 10 min; and preservation at 4°C. The size of the PCR products was determined by 1% agarose gel electrophoresis. Target bands were recovered using an agar DNA gel recovery kit (TaKaRa, Dalian, China). The purified PCR products were connected with the pMD-19T vector, incubated at 25°C overnight for 18 h, and transferred to *E. coli* DH5α receptor cells. Positive recombinants were screened and confirmed using sequencing.

### Sequence alignment and evolutionary analyses of BrCUC2 proteins from different plant species

Bioinformatics was used to predict the molecular properties of the protein encoded by the *BrCUC2* gene. We used ProtParam’s Expasy software (https://web.expasy.org/protparam/) to predict protein physicochemical properties, ProtScale (Expasy - ProtScale) (Wilkins et al., 1999) to analyze hydrophobicity, SignaIP (https://services.healthtech.dtu.dk/services/SignalP-5.0/) (Teufel et al., 2022) to predict signal peptides, TMHMM (https://services.healthtech.dtu.dk/services/TMHMM-2.0/) (Krogh et al., 2001) to predict transmembrane domains, SMART tool to predict conserved structural domains, SOPMA(https://npsa-prabi.ibcp.fr/cgi-bin/npsa_automat.pl?page=npsa%20_sopma.html) (Geourjon and Deléage, 1995) and SWISS-MODEL (Waterhouse et al., 2018) (https://swissmodel.expasy.org/) to predict protein secondary and tertiary structures.

Download the homologous gene sequence of *BrCUC2* from NCBI database. Determine and visualize the distribution position of *BrCUC2* homologous gene on chromosome with TBtools software. Utilizing online software Gene Structure Display Server (GSDS)(Guo et al., 2007) (http://gsds.gao-lab.org/index.php) to analyse the gene structure of *BrCUC2* homologous gene in *B. rapa*. Based on the location information of the *BrCUC2* homologous gene on chromosomes, the chromosome location map of the CUC genes were drawn using by MapChart software (Voorrips, 2002). The conservative motifs of homologous gene were predicted by MEME (https://meme-suite.org/meme/tools/meme) (Bailey et al., 2009). and the protein sequences of different species of *Brassica napus*, *Arabidopsis lyrate*, *Asparagus officinalis*, *Arabidopsis thaliana*, *Brassica oleracea*, *Camelina sativa*, *Glycine max*, *Medicago truncatula*, *Nicotiana attenuata*, *Raphanus sativus*, *Ricinus communis*, *Sesamum indicum* and ‘Longyou 7’ were obtained by NCBI database BLAST comparison, The amino acid sequences were compared by DNAMAN software. Neighbor–joining method was used to construct the phylogenetic tree by MEGA7.0, and the value of Bootstrap method was 1000 (Hall, 2013; Kumar et al., 2016).

### Real-time fluorescence quantitative PCR

RNA was extracted from the leaves, roots, stems, and growth cones of winter *B. rapa* ‘Longyou 7’ and ‘Longyou 99’ after low-temperature treatment and reverse-transcribed into cDNA using a real-time fluorescence quantification biotechnology kit (TaKaRa, Dalian, China) according to the manufacturer’s instructions. The specifically designed primers are listed in **Table 1**. RT-qPCR was performed by adding a 20 μL system containing the target gene and reference gene to a 96–well plate using a fluorescence quantitative PCR apparatus. The reaction was repeated three times, and the reaction procedure was determined according to the manufacturer’s instructions for the specific reagent. The relative gene expression was calculated using the 2^–ΔΔCT^ method (Livak and Schmittgen, 2001).

**Table 1:** Quantitative real-time PCR primer sequences.

Primer	Sequence 5’- 3’
CUC2-1	CCATCGCAGAGGTTGATCTT
CUC2-2	TCTCAGTCCCGTCGGATATT
Actin-F	TGTGCCAATCTACGAGGGTTT
Actin-R	TTTCCCGCTCTGCTGTTGT

### Subcellular localization of BrCUC2

Using the coding sequence (CDS) amplification product of *BrCUC2* as a template, Gateway technology (Liang et al., 2013) was used to amplify the product using aatB joint-specific primers, and the amplified product was cloned into the pDNOR entry vector *via* the BP reaction. After the LR reaction, the gene was introduced into the pEarlygate101-GFP vector, and the constructed vector plasmid was transferred into *Agrobacterium* GV3101 using the electrical conversion method. Bacterial fluid was collected and resuspended in 1 mL of infiltration buffer, and set the optical density (OD) at 600 nm of the solution to 0.6. The lower epidermis of *N. Benthamiana* leaves was labeled with the bacterial solution using a sterile needle tube and then incubated in a 22°C culture room for 48 h. Labeled *N. Benthamiana* leaves were observed and photographed under a laser confocal microscope to determine the location of the genes.

### Self-activation validation of BrCUC2

The constructed positive control plasmids pGBKT7-53+pGADT7-T, negative control plasmids pGBKT7-Lam+pGADT7-T, and pGBKT7-CUC2+pGADT7 plasmids were transformed into yeast strain Y2HGold, respectively. The transformed strains were cultured on DDO/X(SD/-Leu/-Trp/-X-α-gal) medium and incubate in a 30°C incubator for 3–5 days. Select single colonies on QDO/X/A(SD/Leu/-Trp/-His/-Ade/-X-α-gal/AbA) medium. Observe the self-activation phenomenon of pGBKT7-CUC2 based on the growth status of yeast.

### Promoter cloning and cis-acting regulatory element analysis

We searched the region approximately 2000 bp upstream of the *BrCUC2* sequence in the genome of *B. rapa* and designed specific primers using Primer Premier 5.0 software. The following primers were added to the Gateway vector connector sequence: *CUC2* F(5’-3’): AAAAAAGCAGGCTTCCAATATGACACTAATTATGC; *CUC2* R(5’-3’): AGAAAGCTGGGTCGAAGAACTGATGTTAAAAC. Amplification based on the Gateway technique was performed in the following PCR reaction system (total 25 μL): Prime STAR DNA polymerase, 0.25 μL; 5×PrimeSTAR buffer, 5 μL; 2.5 mM dNTP, 1 μL, forward and reverse primers, each 1 μL; template DNA, 2 μL; and ddH_2_O, 14.75 μL. The PCR reaction procedure was as follows: pre-denaturation at 98°C for 10 s and a total of 30 cycles of denaturation at 98°C for 10 s and extension at 68°C for 1 min. The PCR reactions were performed in two rounds, and after two rounds of PCR, the target bands were cut and recovered, connected to the PMD19-T vector, and transformed into *E. coli* DH5α. The promoter sequences were then sequenced after PCR detection of the bacterial solution. *BrCUC2* promoter sequences were analyzed using the PlantCARE database (Lescot et al., 2002) to investigate cis-acting regulatory elements.

### Genetic transformation and β-glucuronidase staining in *A. thaliana*

The constructed plant overexpression vector pEarlyGate101 was transformed into *Agrobacterium* GV3101 using the freeze–thaw method (Weigel and Glazebrook, 2006). When *A. thaliana* grew to the flowering stage, the *Agrobacterium* solution was activated and resuspended in a 5% sucrose solution. The absorbance at 600 nm was adjusted to 0.8, and 3/10000 surfactant (Silwet L-77) was added for later use. Culturing was performed in the dark at 25°C for 24 h and then in the light. The herbicide Glufosinate Ammonium (Basta) (1/10000) (Sangon Biotecch, shanghai, China) was sprayed for screening. After maturation, *A. thaliana* seeds were harvested from each plant.

Resistant *A. thaliana* plants were placed in a GUS staining solution, and wild-type *A. thaliana* was used as the control. The plants were kept at 37°C overnight, rinsed and soaked with 70% ethanol, and photographed under a microscope to observe GUS staining (Liu et al., 2017).

### Screening, identification, and functional analysis of transgenic *A. thaliana*

After vernalization, the first generation T0 transgenic *A. thaliana* seeds were planted in sterilized nutrient soil, sprayed with Basta (10%) two weeks later for screening, and sprayed again three times a week. The surviving *A. thaliana* plants were harvested from the T1 generation seeds. The T1 generation seeds were seeded in 1/2 MS medium containing Basta and screened according to the 3:1 principle. T2 generation seeds were harvested after transplantation. The T2 generation seeds were planted in 1/2 MS medium containing Basta, and all surviving plants were transgenic homozygous plants. Subsequent experiments were conducted after transplantation. Genomic DNA of transgenic *A. thaliana* was amplified using the primers CUC2 F(5’-3’): CTATCCTTCGCAAGACCTTC and CUC2 R(5’-3’): TAATTCCATACGCAATCAAGT and identified *via* PCR and RT-qPCR analyses.

Phenotypic changes were observed in the third-generation transgenic lines and wild-type *A. thaliana* during the seedling, flowering, and mature stages. After four weeks of growth, they were exposed to -4°C for 3, 6, 12, and 24 h and then transferred to room temperature (25°C) to recover growth for seven days, and their survival rate was counted. *A. thaliana* leaves were collected at different treatment times, and their physiological indicators and gene expression levels were measured. Growth at room temperature (25°C) was used as a control. Superoxide dismutase (SOD) activity was determined using the nitroblue tetrazolium (NBT) photoreduction method (Durak et al., 1993). peroxidase (POD) activity was determined using the guaiacol method (Senthilkumar et al., 2021). Proline (PRO) content was measured using the acid ninhydrin method (Abrahám et al., 2010).

## Results

### Identification and analysis of CUC2 gene family in winter *B. rapa*

**Figure 1:**
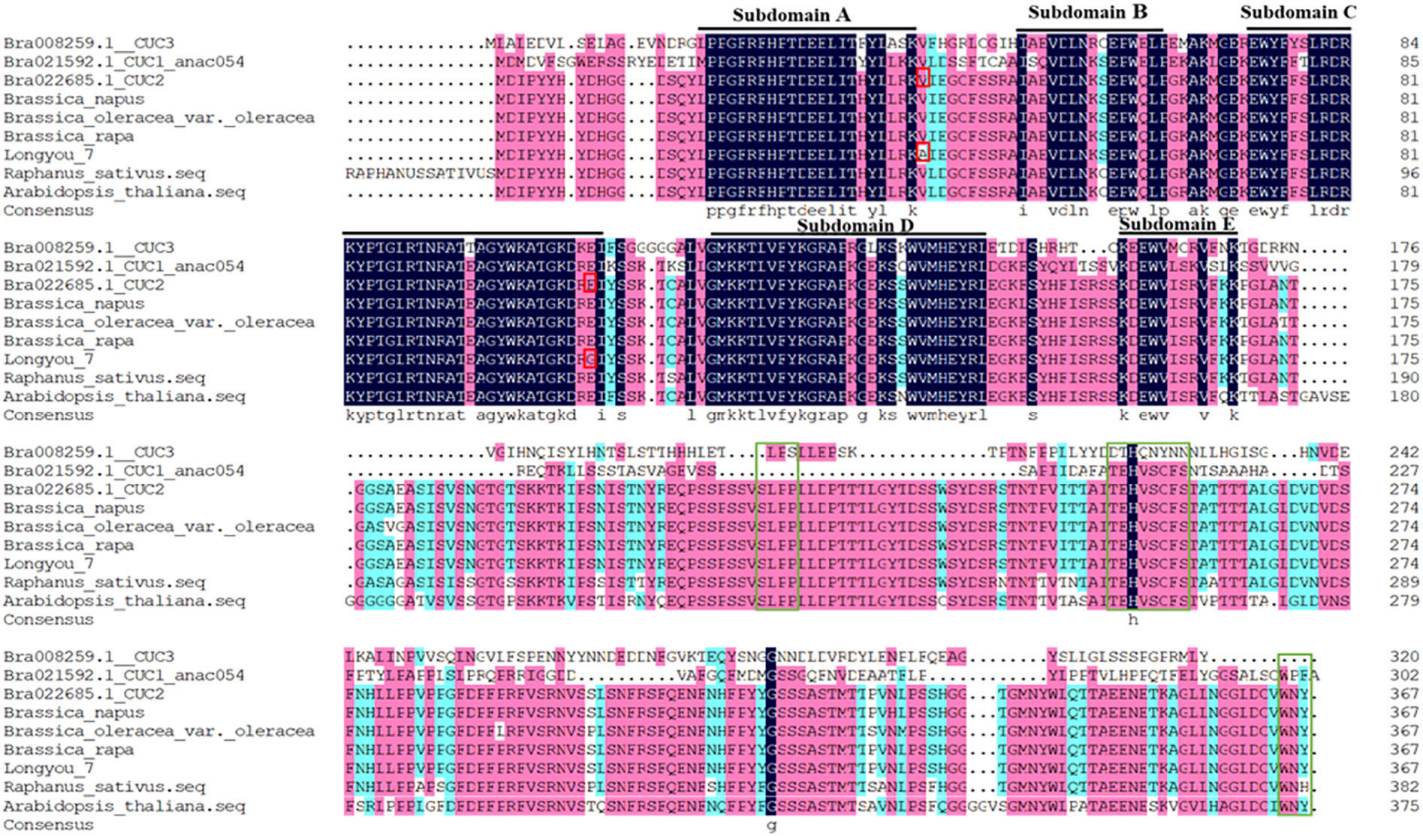
Amino acid sequence comparison of CUC2 protein in different species and homologous protein CUC. Highly conserved subdomains **(A–E)** are indicated by black lines. Amino acid mutation sites are indicated by red boxes. The L motif, V motif and W motif of C-terminal domain are represented by green boxes.

The NAC (PF02365) domain of CUC2 was determined using the Pfam database. Ten CUC genes of *B.rapa* were selected, and the homologous BrCUC2 protein sequences of different species were screened by BLAST-Protein (BlASTP) tool. DNAMAN software was used to compare the protein sequences of BrCUC2 and its homologous genes *CUC1*、*CUC3*, and *BrCUC2* (*B.rapa*, *B.napus*, *B.oleracea*, *R.sativus, A. thaliana*) in different species (**Figure 1**). The results showed that all CUC protein sequences contained a highly conserved NAC domain at the N-terminus, including A, B, C, D and E subdomains. the subdomains A, C and D were highly conserved, while the subdomains B and E were structurally variable and might be involved in different biological functions. The integrity of the NAC domain is essential for CUC2 to promote the formation of adventitious buds in callus tissue (Taoka et al., 2004). The absence of any of the five motifs affects the formation of adventitious buds. The C-terminal domain of CUC2 protein contains three conserved motifs, namely the V motif (TEHVSCFS), L motif (SLPP), W motif (WNY), and two serine rich regions (S). Among them, the W motif is essential for the transactivation of the CUC2 protein, and the V motif contains miRNA binding sites, which is responsible for regulating *CUC2* gene activity. The intact CUC2 protein plays an important role in Shoot apical meristem (SAM) formation or plant organ separation. The BrCUC2 (Bra022685) protein sequence of *B.rapa* was 99.46% similar to the CUC2 protein of ‘Longyou 7’, and there were two amino acid mutation sites, alanine (A) of ‘Longyou 7’ was changed to valine (V), glycine (G) was changed to glutamic acid (E).The homology was 89.01% with *Raphanus sativus* (*R. sativus*), 97.28% with *Brassica oleracea* (*B. oleracea*) and 99.46% with *Brassica napus* (*B. napus*). Suggesting that the BrCUC2 (Bra022685) protein sequence is highly conserved during evolution process.

**Figure 2:**
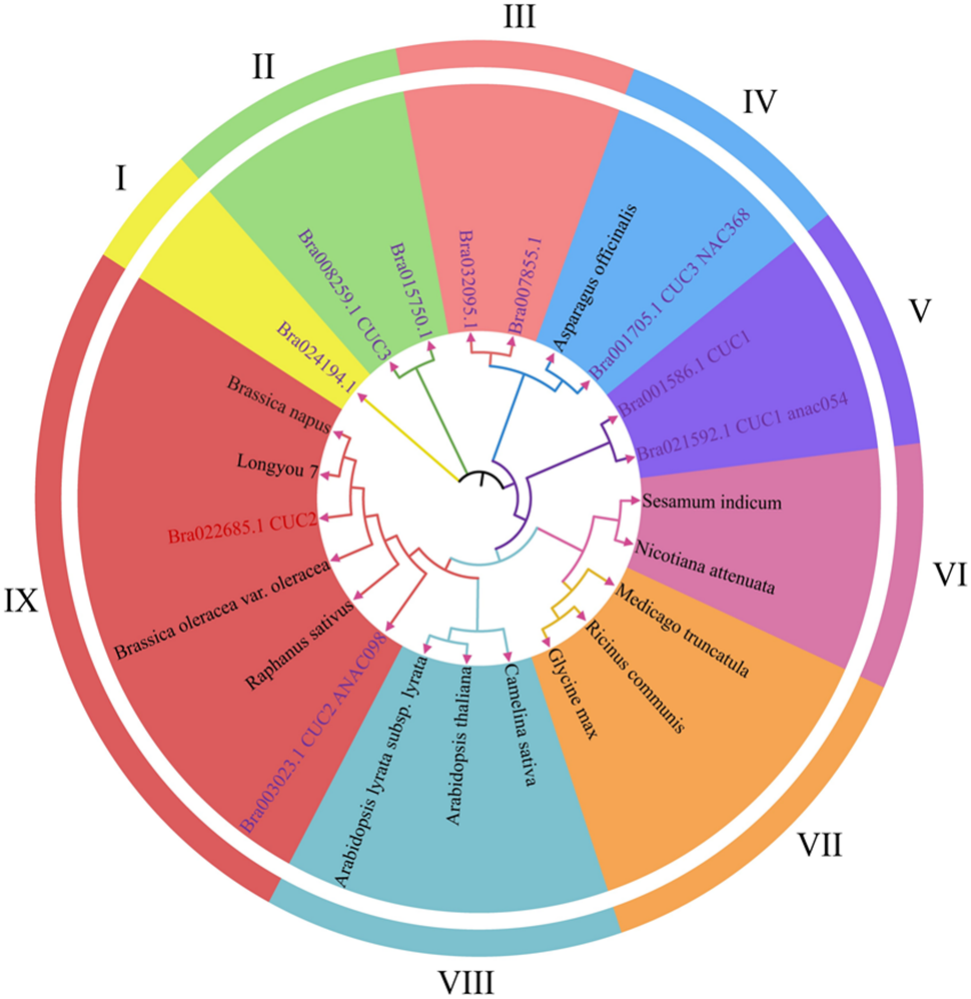
Evolutionary analysis of CUC2 in different species. The 9 subfamilies have different colors. Purple font represents members of the *BrCUC2* homologous genes, The others are *BrCUC2* in different species.

In order to further clarify the functional and evolutionary relationships of the *BrCUC2* gene in the growth cone of winter *B.rapa*, MEGA11 was used to construct a phylogenetic tree, and the evolutionary relationships were compared between CUCs and BrCUC2 proteins in 13 different species (including *B. napus*, *B. rapa*, *C. sativa*, *B. oleracea*, *R. sativus*, etc.) (**Figure 2**). The results showed that they were mainly divided into nine subfamilies, among which BrCUC2 (Bra002685.1) was more closely related to *B. oleracea*, *B. napus*, *R. sativus* and Bra003023. CUC1 (Bra001586) and CUC3 (Bra008259) were closely related to *Asparagus officinalis* (*A. officinalis*). It is speculated that the *BrCUC2* gene in winter *B. rapa* ‘Longyou 7’ is functionally similar to that in *B. napus*, *B. oleracea* and *R. sativus*.

### Chromosome mapping and structure analysis of CUCs gene in winter *B. rapa*

**Figure 3:**
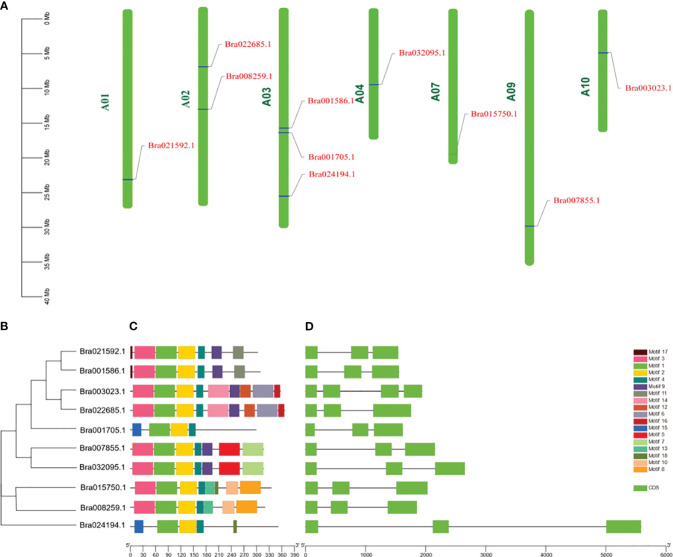
Chromosomal localization and structural analysis of the CUCs gene. **(A)** Chromosomal location distribution of CUCs genes. **(B)** Phylogenetic relationships of CUCs genes. **(C)** Schematic representation of conserved motifs in CUCs proteins. Different colored boxes represent different patterns. The black lines represent non-conserved sequences. **(D)** CUCs exon/intron structure. The green boxes represent exons; The space between boxes is the intron. The scale bars at the bottom of **(C, D)** represent the lengths of genes and motifs, respectively.Cloning and Sequence Analysis of the *BrCUC2* (Bra022685) Gene.

Ten CUC genes in *B. rapa* were distributed on seven chromosomes (**Figure 3A**), two of which were distributed on chromosome A02, three on chromosome A03, and the rest on chromosomes A01, A04, A07, A09 and A10, respectively. The positions of introns and exons were determined by comparing the genomic DNA with the full-length cDNA of CUCs (**Figure 3D**). the results showed that Bra003023 contained three introns and the rest nine genes contained two introns. Ten CUC genes contained a total of 18 motifs, and all contained motif 1, motif 3 and motif 4, of which Bra022685 and Bra003023, Bra007855 and Bra032095, Bra021592 and Bra001586 contained the same motif, respectively. and the same subfamily of CUC proteins in the phylogenetic tree had the same motif, indicating that they were highly conserved (**Figures 3B, C**, **S1**; **Table S1**).

The BrCUC2 gene (1104 bp) was amplified using the cDNA template from the growing site of ‘Longyou 7,’ and the result was consistent with the predicted fragment size. The BrCUC2 gene sequence encodes 367 amino acids. The theoretical isoelectric point (PI) was 8.66, fat index was 58.71, instability index was 44.07, and total average hydrophilic value was -0.569. The BrCUC2 protein was predicted as an unstable hydrophilic protein (**Figure S2A**). No transmembrane regions and signaling peptides were detected (**Figures S2B, C**). The conserved domain was distributed among 18–144 amino acids and composed of 127 amino acids, and the conserved sequences belonged to the NAM superfamily (**Figure S1**). The secondary and tertiary structures of the BrCUC2 protein mainly consisted of irregular curling (65.12%), extended chain (16.25%), α helix (14.17%), and β angle (4.36%) (**Figures S2D, E**).

### Prediction and functional analysis of cis-acting regulatory elements in the *BrCUC2* promoter region

**Figure 4:**
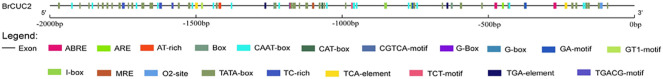
Promoter cis-acting regulatory element distribution. Different colored rectangular boxes represent individual cis-acting components.

Promoters are important gene elements that regulate gene expression. By analyzing the cis-acting elements, the pathway genes that may participate in abiotic stress-tolerance mechanisms can be better understood (Hernandez-Garcia and Finer, 2014). Using the genomic DNA of the ‘Longyou 7’ growth cone of winter *B. rapa* as a template, the *BrCUC2* promoter sequence with a length of approximately 2000 bp was amplified and sequenced after connecting to the vector.The sequencing was correctly used to prepare *Agrobacterium* liquid transformed *Arabidopsis*. PlantCARE and GSDS2.0 online software were used to analyze the *BrCUC2* promoter sequence and predict the cis-acting regulatory elements (**Table 2**; **Figure 4**). The results showed that the promoter region of this gene contains ABRE-acting elements involved in abscisic acid and stress responses, TGA elements involved in the auxin response, CAT-box elements involved in meristem expression. Tc-rich repeats are involved in stress response; ARE active elements are involved in anaerobic responses; Box4, GA-motif, GT1-motif, I-box, and TCT-motif active elements are involved in light response; and several CAAT-box and CAT-box elements are core promoters.

**Table 2:** Cis-acting elements and their functions in the *BrCUC2* promoter region.

Cis-acting element	Core sequence	Position	Function
ABRE	ACGTG	﹣417,﹣1522,﹣1724	Involved in the abscisic acid responsiveness
ARE	AAACCA	1548	Essential for the anaerobic induction
AT-rich sequence	TAAAATACT	578	Element for maximal elicitor-mediated activation
Box 4	ATTAAT	766, ﹣1567, ﹣1373	Light responsiveness
CAAT-box	CAAT	5, -1831	Common cis-acting elements in promoter and enhancer regions
CAT-box	GCCACT	930	Related to meristem expression
CGTCA-motif	CGTCA	419	Involved in the MeJA-responsiveness
GA-motif	ATAGATAA	-1239	Light-responsive element
GT1-motif	GGTTAAT	518	Light-responsive element
I-box	GATAAGGTG	-1237	Light-responsive element
O2-site	GATGATGTGG	1883	Involved in zein metabolism regulation
TC-rich repeats	GTTTTCTTAC	248	Involved in defense and stress responsiveness
TGA-element	AACGAC	﹣735,﹣1358	Auxin-responsive element
TCT-motif	TCTTAC	1032	Light-responsive element

### Subcellular localization and self-activation validation of BrCUC2

**Figure 5:**
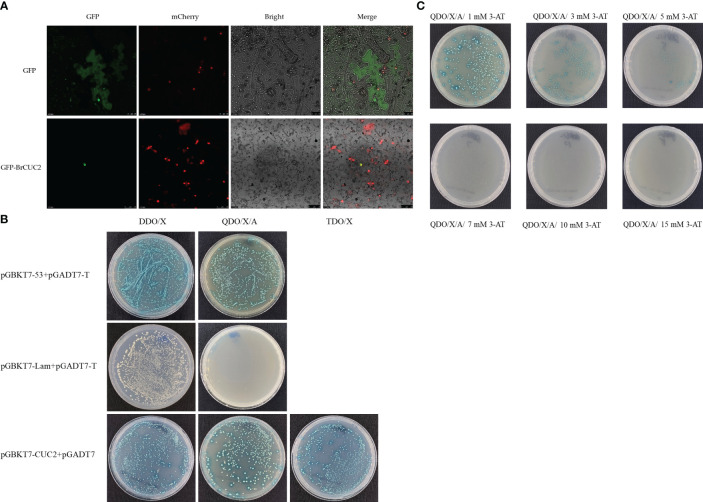
**(A)** Subcellular localization of PEarlyGate101-BrCUC2-GFP protein, empty GFP as a control, mCherry, a nuclear localization protein. **(B)** Self-activation verification of BrCUC2. The positive control pGBKT7-53+pGADT7-T, the negative control pGBKT7-Lam+pGADT7-T, and the experimental group pGBKT7-CUC2+pGADT7 were transferred to yeast strain Y2HGold, respectively. Cultured on DDO/X (SD/-Leu/-Trp/- X-α-Gal) and QDO/X/A (SD/Leu/-Trp/-His/-Ade/-X-α-Gal) medium, respectively. TDO/X (SD/-Leu/-Trp/-His/-X-α-Gal). **(C)** Screening of 3-AT inhibition at different concentrations in QDO/X/A medium. 3-amino-1,2,4-triazole(3-AT), 3-AT is a competitive inhibitor of histidine and can inhibit mild self-activation.

The fusion expression vector *PEarlyGate101 BrCUC2* GFP was constructed using Gateway technology, and tobacco leaves were transformed instantaneously with empty GFP as a control. After 48 h of growth in the dark, scanning and photography were performed using a confocal laser microscope. The results showed that the *BrCUC2* GFP fusion protein was distributed in the nucleus (**Figure 5A**).

To further characterize the function of BrCUC2, we investigated whether BrCUC2 has self-activating activity in yeast cells. The positive control pGBKT7-53+pGADT7-T, negative control pGBKT7-Lam+pGADT7-T, and pGBKT7-CUC2+pGADT7 were transformed into Y2HGold yeast cells and cultured on DDO/X (SD/-Leu/-Trp/-X-α-gal) and QDO/X/A (SD/-Leu/-Trp/-His/-Ade/-X-α-gal/AbA) medium, respectively. The results showed that the positive control combination grew and turned blue on DDO/X and QDO/X/A medium, while the negative control group grew but remained blue on DDO/X media, and did not grow on QDO/X/A media, indicating the success of the positive control and negative control experiments. The experimental group pGBKT7-CUC2+pGADT7 grew on DDO/X medium, grew and turned blue on both TDO/X (SD/-Leu/-Trp/-His/-X-α-gal) and QDO/X/A media (**Figure 5B**), indicating that the pGBKT7-CUC2 plasmid was successfully transferred into the yeast strain and showed self-activation in the yeast strain. Further inhibition experiments were conducted using (3-amino-1,2,4-triazole)3-AT, and the results showed that the growth turned blue on QDO/X/A/(1-5) mM 3-AT and weakened with the increase of 3-AT concentration. However, it did not grow on QDO/X/A/(7-15) mM 3-AT medium (**Figure 5C**), indicating that the QDO/X/A/7 mM 3-AT screening conditions can inhibit the self-activation phenomenon of pGBKT7-CUC2.

### Analysis of *BrCUC2* gene expression

**Figure 6:**
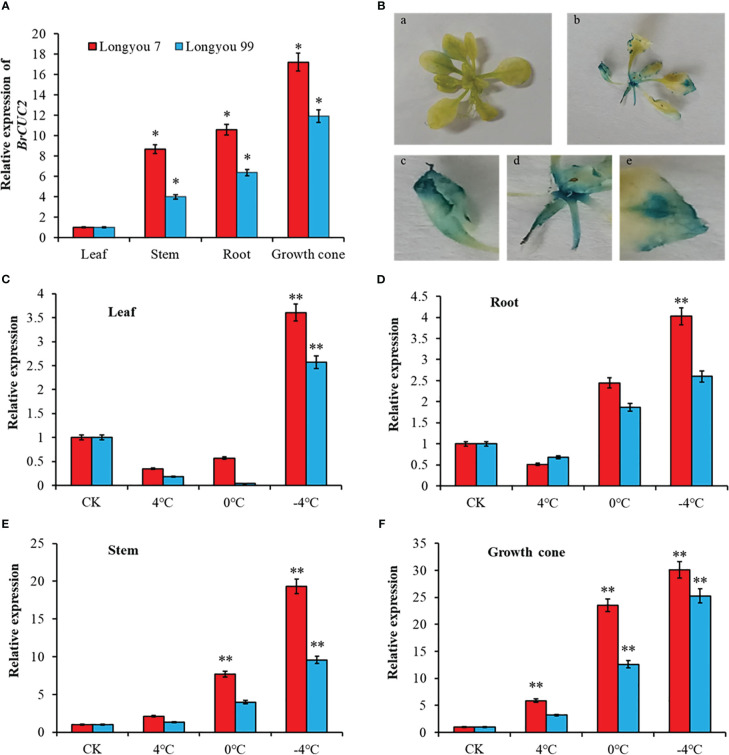
*BrCUC2* tissue expression specificity. **(A)** Expression level of *BrCUC2* in different tissues. **(B)** GUS mapping of *BrCUC2* transgenic *Arabidopsis thaliana*. a, GUS staining of whole wild - type *A. thaliana*; b, GUS staining of whole transgenic *A. thaliana*; c-e, Partial magnification of b **(C)** Expression of *BrCUC2* gene in leaf under low temperature stress. **(D)** Expression of *BrCUC2* gene in Root under low temperature stress. **(E)** Expression of *BrCUC2* gene in Stem under low temperature stress. **(F)** Expression of *BrCUC2* gene in growth cone under low temperature stress. Symbols “*”and “**” indicate significant differences at *P values* of 0.05 and 0.01, respectively.

To clarify the differences in the expression of the *BrCUC2* gene in various winter rapeseed tissues, RT-qPCR was performed to determine the relative expression levels of *BrCUC2* in the leaves, stems, growth cones, and roots (**Figure 6A**). The results showed that the expression of *BrCUC2* in different tissues of ‘Longyou 7’ and ‘Longyou 99’ was significantly different. The expression level in ‘Longyou 99’ was 10.9-times higher in the growth points than in the leaves, whereas that in ‘Longyou 7’ was 16.2-times higher in the growth cone than in the leaves, indicating that the *BrCUC2* gene mainly acts on the growth cone of winter *B. rapa*.

To verify the expression pattern of *BrCUC2*, GUS tissue staining was performed on wild-type and transgenic *A. thaliana* seedlings (**Figure 6B**). The results revealed that the GUS signal was absent in the wild-type *A. thaliana* but present on the leaf edges, stems, and growth points of transgenic *A. thaliana* seedlings, indicating that the BrCUC2 promoter cloned from winter *B. rapa* could drive downstream GUS reporter gene expression with tissue specificity.

To investigate the effect of cold stress on *BrCUC2* expression, ‘Longyou 7’ and ‘Longyou 99’ were treated at 0, 4, and -4°C, RT-qPCR was performed to measure the expression. The results showed differences in the relative expression of the *BrCUC2* gene among different tissues after low-temperature stress. As the temperature decreased, the expression of the *BrCUC2* gene was upregulated and reached the highest value at -4°C. The difference was most significant in stem and growth cone and higher in ‘Longyou 7,’ which presents strong cold resistance and a concave growth cone, than in ‘Longyou 99,’ which presents weak cold resistance and a convex growth cone. In the leaves, under normal temperature control, the expression of *BrCUC2* in ‘Longyou 7’ and ‘Longyou 99’ was downregulated at 4°C and 0°C, and after treatment at -4°C, the expression was 2.6- and 1.5- times higher than those in the control, respectively (**Figure 6C**). In the roots, the expression of *BrCUC2* in ‘Longyou 7’ and ‘Longyou 99’ was downregulated at 4°C and upregulated at -4°C and 0°C, respectively. After treatment at -4°C, the expression was 3.0- and 1.6- times higher than those in the control, respectively (**Figure 6D**). With a decrease in temperature, *BrCUC2* was upregulated in the stem and growth cone compared with that in the control (**Figures 6E, F**). After treatment at -4°C, the expression levels of *BrCUC2* in ‘Longyou 7’ and ‘Longyou 99’ were 18.3- and 8.5-times higher in the stem and 29.1 and 24.3-times higher in the growth cone than those in the control, respectively.

### Screening and identification of the transgenic *BrCUC2* gene in *A.thaliana*

The first generation T0 *BrCUC2*-overexpressing plants were vernalized and planted in nutrient-rich soil. Two weeks later, they were sprayed with a 10% Basta solution. After exposure to the spray three times, seedlings that did not exhibit resistance to the herbicides turned yellow and died. Normal surviving seedlings were transplanted, and seeds were harvested from individual plants to obtain the T1 generation. The T1 generation of *A. thaliana* was selected on 1/2MS medium containing Basta according to the 3:1 principle. Seedlings with normal leaf and root growth were transplanted, and individual plants were harvested to obtain the T2 generation. The T2 generation of *A. thaliana* was screened for all surviving seedlings on 1/2MS medium containing Basta to obtain homozygous transgenic lines, which were then transplanted into pots for subsequent tests (**Figure S3A**).

After extracting *BrCUC2* transgenic *A. thaliana* DNA, PCR amplification results showed no amplification bands in the wild-type *A. thaliana* single plants; however, 10 bands of approximately 1100 bp were amplified from 11 single transgenic plants. Among them, C2 had no bands, indicating that the transformation of the Br*CUC2* gene in C2 single plants failed, while the 10 remaining single plants were successfully transformed, indicating that they were transgenic plants, with C6 and C10 showing lighter bands (**Figure S3B**).

RT-qPCR identification was performed on 10 overexpression plants and wild-type transgenic plants, and the results showed that the *BrCUC2* gene was detected in all overexpression plants. The expression levels varied among different individual plants, with the highest expression levels of the *CUC2* gene in C3 and C9, which were 7.53 and 7.79 times higher than those in the WT (**Figure S3C**).

### Phenotypic differences associated with the *BrCUC2* gene in *A. thaliana* treated at low temperature

**Figure 7:**
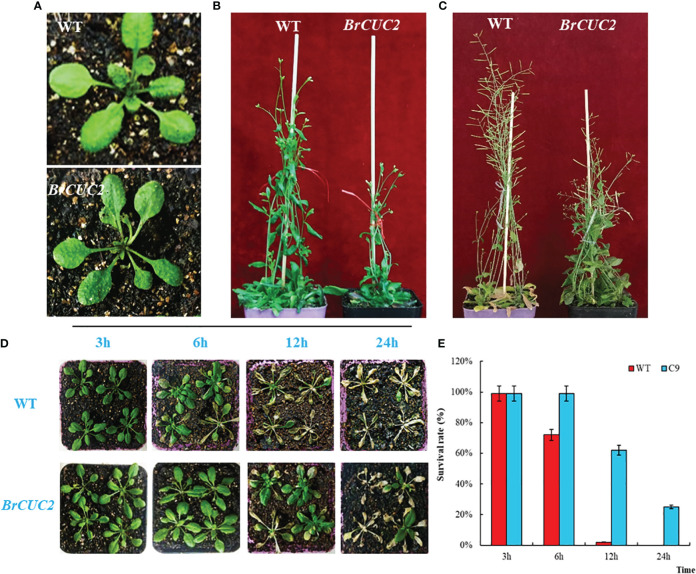
*BrCUC2* transgenic *A*. *thaliana* phenotype. **(A)** Seedling stage, **(B)** Flowering stage, and **(C)** Maturity stage. **(D)** Phenotypes of transgenic and wild-type *A*. *thaliana* after low-temperature treatment at -4°C for 3, 6, 12, and 24 h. **(E)** Survival rate of plants after low-temperature treatment. C9: *BrCUC2* overexpressed plant.

Wild-type and *BrCUC2*-overexpressed *A. thaliana* phenotypes were observed at the seedling (4 weeks old), flowering, and mature stages under the same growth conditions (16h light/8 h dark cycle at 25°C). Comparisons between the wild-type and *BrCUC2*-overexpression *A. thaliana* plants showed that significant phenotype differences did not occur at the seedling stage but did occur at the flowering stage, with weaker growth and development and a dwarf phenotype (**Figures 7A, B**). The overexpression *A. thaliana* lines developed slowly and were set later than the wild-type at the mature stage. When wild-type *A. thaliana* plants were clamped and matured, the lateral branches of overexpression lines were still in the flowering stage, and the seed-setting rate was lower than that of the wild type (**Figure 7C**).

To verify the response of the *BrCUC2* gene to low-temperature stress in plants, 4-week-old wild-type and *BrCUC2*-overexpression plants were treated at -4°C for 3, 6, 12, and 24 h and then returned to room temperature (25°C) for 7 d to observe the phenotypes (**Figures 7D, E**). All plants grew normally after low-temperature treatment for 3 h; however, the survival rate of the wild-type plants was 72% after low-temperature treatment for 6 h and decreased to 8% after 12 h, with most of the leaves dying. After low-temperature treatment for 6 h, the survival rate of *BrCUC2*-overexpression plants was 99%; after 12 h, some leaves yellowed, and the survival rate was 62%, and after 24 h, the survival rate was 25%. These results indicate that *BrCUC2* overexpression enhances plant low-temperature tolerance over a short period.

### Physiological parameters of *BrCUC2* gene transfer in *A. thaliana*

**Figure 8:**
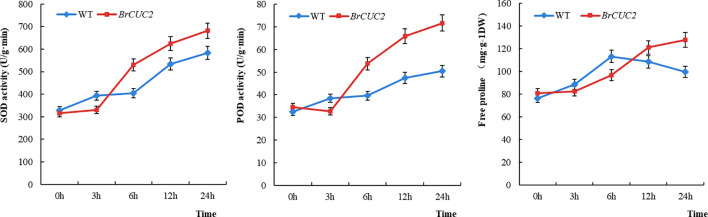
Physiological indices SOD、POD、Proline of *Arabidopsis thaliana* overexpressing *BrCUC2* gene at low temperature. WT: wild type, *BrCUC2*: overexpressed plant.

The physiological indicators of wild-type and overexpressed *A. thaliana* after low-temperature treatment were analyzed after treatment at -4°C. The enzyme content in both plants gradually increased over time. The superoxide dismutase(SOD)activity、peroxidase (POD) activity and proline content in the *BrCUC2*-overexpressing plants were higher than those in the wild-type plants, reaching the highest values after 24 h (16.9%, 41.9%, and 22.9% higher than those in the wild-type, respectively) (**Figure 8**). This result indicates that the *BrCUC2* gene is involved in the response of winter *B. rapa* to low-temperature stress.

## Discussion

The *A. thaliana* NAC family transcription factor *CUC2*, a negative regulator of plant growth (Takada et al., 2001), plays an important role in the formation of apical meristems and organ margins, leaf edge morphological formation, growth, and development. The formation of meristem edges in *A. thaliana* is mediated by the NAC family transcription factors *CUC1*, *CUC2*, and *CUC3* (Bertrand et al., 2003; Kamiuchi et al., 2014). The *CUC1* and *CUC2* are negatively regulated by *miR164 *(Bhatt et al., 2004). Kwon et al (Kwon et al., 2006) found that the *CUC2* gene could fuse cotyledons and flower organogenesis in *A. thalia*na. Souer et al (Souer et al., 1996) showed that *CUC2* is highly homologous to petunia genes necessary for flower organ development, cotyledon separation, and apical meristem embryogenesis. In *A. thaliana*, the *CUC2* gene is necessary for the activation of the *STM* gene during embryonic development (Lenhard et al., 2002). Studies have shown that *STM* can directly activate *CUC2* (Scofield and Murray, 2006), and mutations in the *CUC2* gene can change the cotyledon traits of *A. thaliana* and cause developmental defects in its apical meristem (Vroemen et al., 2003). *STM* or *CUC2* gene mutations affect the number of plant stem cells and lead to cotyledon fusion (Mallory et al., 2004). However, the regulatory mechanism of the *CUC2* gene in winter rapeseed growth sites has not yet been reported.

In the present study, the *BrCUC2* gene was cloned from the growth point of winter *B. rapa*, which controls the growth point differentiation of *A. thaliana*. The *BrCUC2* gene was expressed in the leaves, stems, roots, and growing points of winter *B. rapa*, with the highest expression level observed at the growing points. The relative expression level was as follows: the expression level of *BrCUC2* in the growing point of ‘Longyou 99’ was 10.9 times that in the leaves, and the expression level of *BrCUC2* at the growing point of ‘Longyou 7’ was 16.2 times that in the leaves. After 72 h of low-temperature treatment, the expression in the leaves was initially downregulated and then upregulated, while the expression in roots, stems, and growth points was upregulated and reached the highest value at -4 °C. The differences were most significant in the stems and growth points. The expression in ‘Longyou 99,’ which presents weak cold resistance and a raised growth point, was lower than that in ‘Longyou 7,’ which presents strong cold resistance and a depressed growth point. These results indicate that the *BrCUC2* gene might be induced by low-temperature stress and inhibit the growth of growing sites. Moreover, the high *BrCUC2* content caused the ‘Longyou 7’ growth point to sag and grow below the ground.

Bioinformatics analysis and functional verification showed that the *BrCUC2* gene contains a conserved NAM superfamily domain, *CUC2* is a transcription factor in the NAC family, and NAC domain proteins usually contain two relatively independent domains, one is a highly conserved N-terminal domain, divided into five (A-E) sub domains, responsible for DNA binding. The C-terminal region is highly divergent and serves as a transcriptional activation region, which endows NAC protein with functional variations (Bian et al., 2020). By comparing the protein sequences of BrCUC2 in different species and its homeotic genes, we found that the N-terminal and C-terminal of BrCUC2 contain NAC domains. Systematic evolution analysis shows that BrCUC2 is closely related to *B. oleracea*, and the CUCs gene contains similar conserved motifs, indicating that functional similarity was maintained during evolution.

Cloning the *BrCUC2* promoter sequence at the growth site of winter *B. rapa* showed that *BrCUC2* contained meristem-forming elements, indicating that the expression of *BrCUC2* would affect the formation of the meristem. The *BrCUC2* gene mainly present in the nucleus, and yeast self-activation verifies its self-activation activity, which may acts as a transcription factor. The *BrCUC2* promoter contains various photoresponsive elements, hormone- and stress-responsive elements. When plants are subjected to low temperature stress, transcription factors activate the expression of low-temperature -responsive genes by binding cis-acting elements on the gene promoter, thus regulating the signaling pathways in the plant to improve low temperature tolerance. The bZIP transcription factor binds to the ABRE element (ACGT) on the target gene promoter to regulate the expression of downstream low temperature response genes (Hwang et al., 2016). CsbZIP6 is a negative regulatory factor for low temperature stress response in tea plants, leading to a downregulation of downstream low temperature response gene expression and affecting the sensitivity of tea plants to ABA (Wang et al., 2017). The *Arabidopsis* gene AtbZIP1 binding ABRE action element and regulates plant response to low temperature stress through ABA-dependent signal transduction pathway (Sun et al., 2011b). *BrCUC2* promoter contains ABRE, ARE, CGTCA-motif, TC-rich repeats and TGA-element elements, which may be involved in the regulation of low temperature stress response. GUS is mainly observed in the leaf margins, stems, and meristems. Research on the expression pattern of the *BrCUC2* gene in winter rapeseed under low-temperature stress and GUS tissue staining showed that the light response elements, meristem formation, and stress-related elements contained in *BrCUC2* played an important role, which is consistent with previous research results on the *CUC2* promoter of *B. platyphylla*.

The natural environment is an important factor influencing plant growth and development. To ensure normal growth, plants must change their molecular and physiological mechanisms in response to external stress. SOD and POD, as scavengers of reactive oxygen species (ROS), can effectively protect plants from low temperatures and other abiotic stresses (Nadarajah, 2020).

Zhang et al (Zhang et al., 2020) found that overexpression of the *SikRbcs2* gene significantly increased SOD, POD, and CAT enzyme activity in tomato *SikRbcs2* transgenic *tobacco* under 4°C stress. The transgenic tomato *ZPR1* gene significantly enhanced *Arabidopsis* physiological activity and cold resistance (Li et al., 2012). The *cuc1-2* double mutant of *A. thaliana* has an abnormal stem end meristem tissue formation (Aida et al., 1997; Uauy et al., 2006). The plant transformed from *B. platyphylla* to 35S::BPCUC2 was shorter than that of the normal plant (Liu et al., 2018). This phenomenon may be due to *CUC2* overexpression, which affects plant growth by affecting the growth of plant meristems. In this study, we found no difference in the phenotypes of the wild-type and *CUC2*-overexpression strains at the seedling stage, whereas the growth characteristics of the overexpression plants were weaker and shorter during the flowering and maturation stages. This finding indicates that the *BrCUC2* gene, as a negative regulatory factor for plant growth, has an inhibitory effect on the growth cones of winter *B. rapa*. After low-temperature treatment, the physiological activity of the overexpression plants was significantly higher than that of wild-type *A. thaliana* only at 6 and 12 h, and there was no difference after 24 h, indicating that the *BrCUC2* gene can improve the low-temperature tolerance of transgenic *A. thaliana* over a short period.

## Conclusion

The *BrCUC2* gene was cloned from the growth point of winter *B. rapa*. It encodes 367 amino acids, and the conserved sequence belongs to the NAM superfamily. Multiple sequence alignment revealed that the N-terminal amino acid sequence of BrCUC2 homologous CUC proteins contained a highly conserved NAC domain. Systematic evolution analysis showed that *BrCUC2* in winter *B. rapa* had the highest affinity for *B. oleracea*, *B. napus*, and *R. sativus*. BrCUC2 is expressed in the nucleus and has self-activition. After low-temperature stress, the expression level of *BrCUC2* was the highest at the growth site. As the temperature decreased, *BrCUC2* was upregulated and presented higher expression in ‘Longyou 7,’ which presents strong cold resistance and a concave growth site, than in ‘Longyou 99,’ which presents weak cold resistance and a protruding growth site. The *BrCUC2* promoter sequence contains a variety of action elements related to light, hormone, and stress responses and meristem expression. GUS chemical tissue staining mainly revealed expression at the edges of the leaves, stems, and growth points in transgenic *A. thaliana*. The overexpression of the *BrCUC2* gene in *A. thaliana* is a characteristic of stunted plant development. After 12 h of low-temperature treatment, some leaves of the *BrCUC2*-overexpression *A. thaliana* withered and yellowed, and the survival rate was 62%; however, the survival rate of wild-type *A. thaliana* decreased to 8%, and most of the leaves died. After 24 h of low-temperature treatment, the physiological indicators SOD, POD, and PRO reached their highest values (16.9%, 41.9%, and 22.9% higher than that of the wild type, respectively). This result indicates that the *BrCUC2* gene can improve the low-temperature tolerance of transgenic *A. thaliana*. This study aimed to explore the regulatory mechanism of *BrCUC2* at the growth site of winter *B. rapa* to provide a foundation for studying cold resistance in winter *B. rapa*.

## Data availability statement

The datasets presented in this study can be found in online repositories. The names of the repository/repositories and accession number(s) can be found in the article/**Supplementary Material**.

## Author contributions

XT: Data curation, Writing – original draft. YZ: Data curation, Writing – review & editing. JW: Data curation, Funding acquisition, Methodology, Writing – review & editing. RZ: Data curation, Writing – review & editing. JJ: Data curation, Writing – review & editing. RL: Data curation, Writing – review & editing. WM: Data curation, Writing – review & editing. YL: Data curation, Writing – review & editing. WW: Methodology, Writing – review & editing. YP: Methodology, Writing – review & editing. GY: Methodology, Writing – review & editing. LL: Methodology, Writing – review & editing. XL: Methodology, Writing – review & editing. LM: Funding acquisition, Methodology, Writing – review & editing. WS: Methodology, Writing – review & editing.

## Supplementary material

Supplementary Figure 1 Conserved motifs logo in CUCs proteins.

Supplementary Figure 2 Structural characteristics of the *BrCUC2* gene.

Supplementary Figure 3 Screening of *BrCUC2* transgenic *Arabidopsis thaliana*.

Supplementary Table 1 Motif prediction of BrCUC2 protein by MEME server.
