# Supplementary figures and images for: Cloning and functional analysis of the BrCUC2 gene in Brassica rapa L
Source: Front Plant Sci. 2023 Oct 30;14:1274567. doi: 10.3389/fpls.2023.1274567 (PMC10642757; doi:10.3389/fpls.2023.1274567)

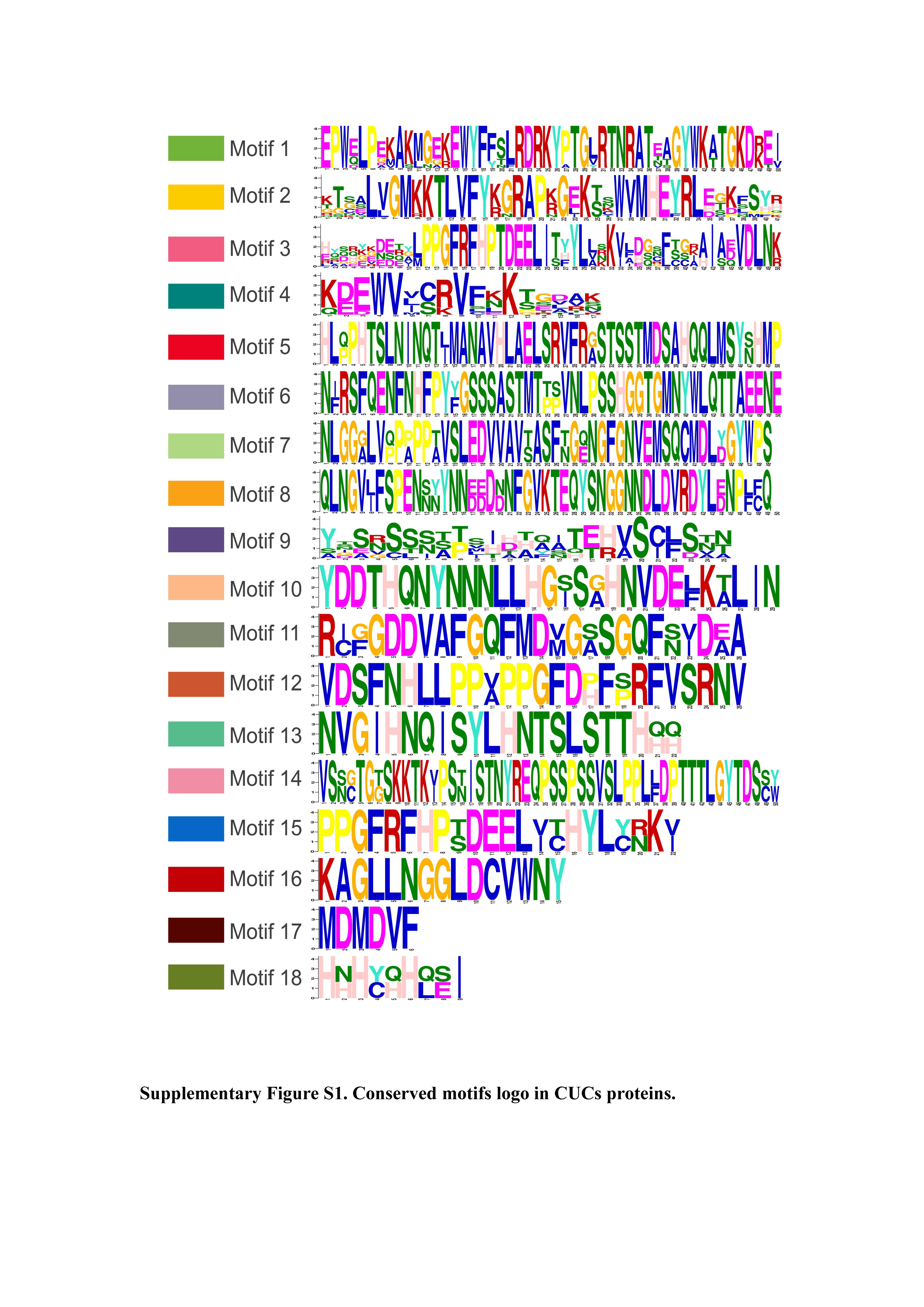

Supplement: Supplementary Figure 1 — Conserved motifs logo in CUCs proteins. [file Image_1.tif]

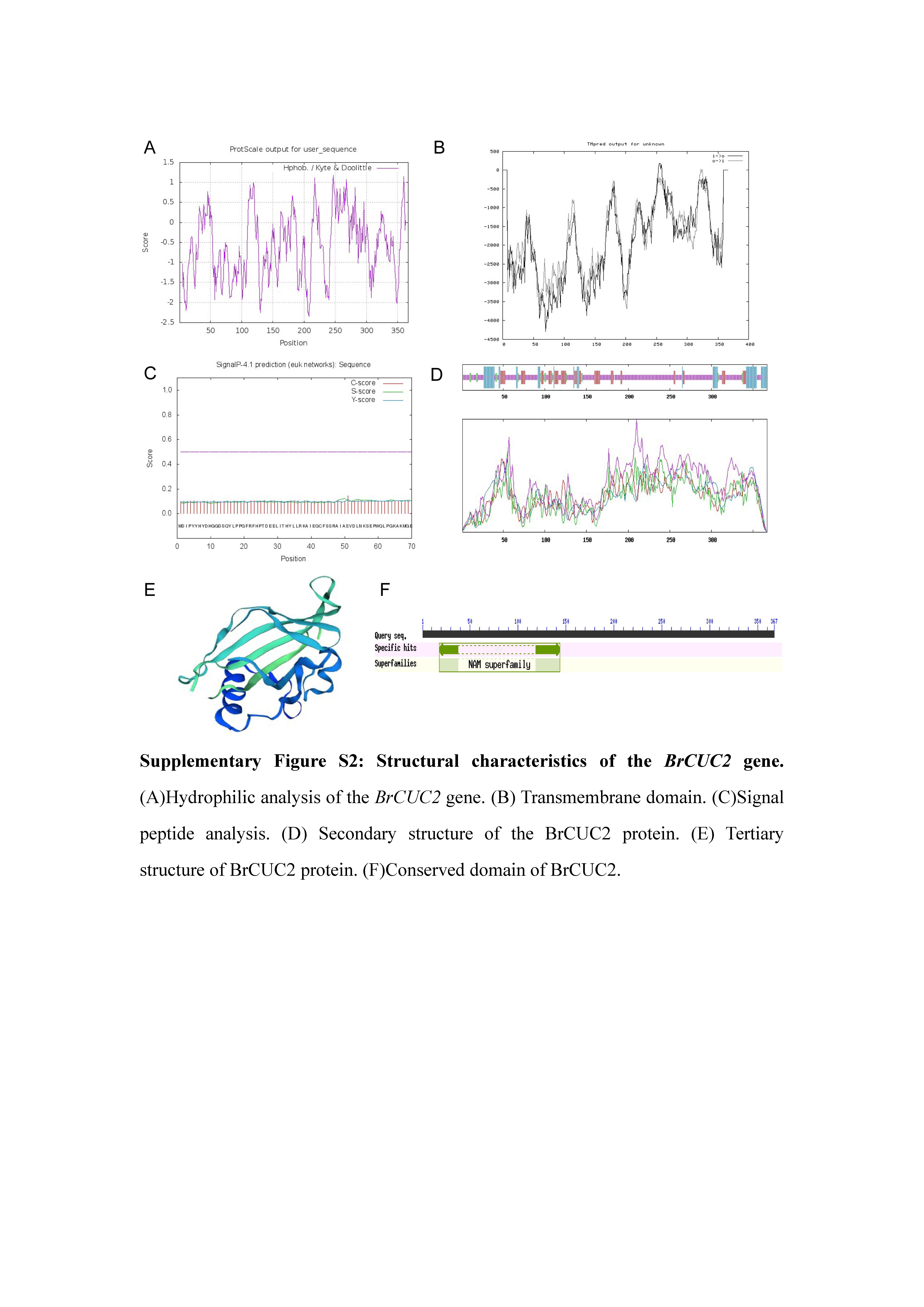

Supplement: Supplementary Figure 2 — Structural characteristics of the BrCUC2 gene. [file Image_2.tif]

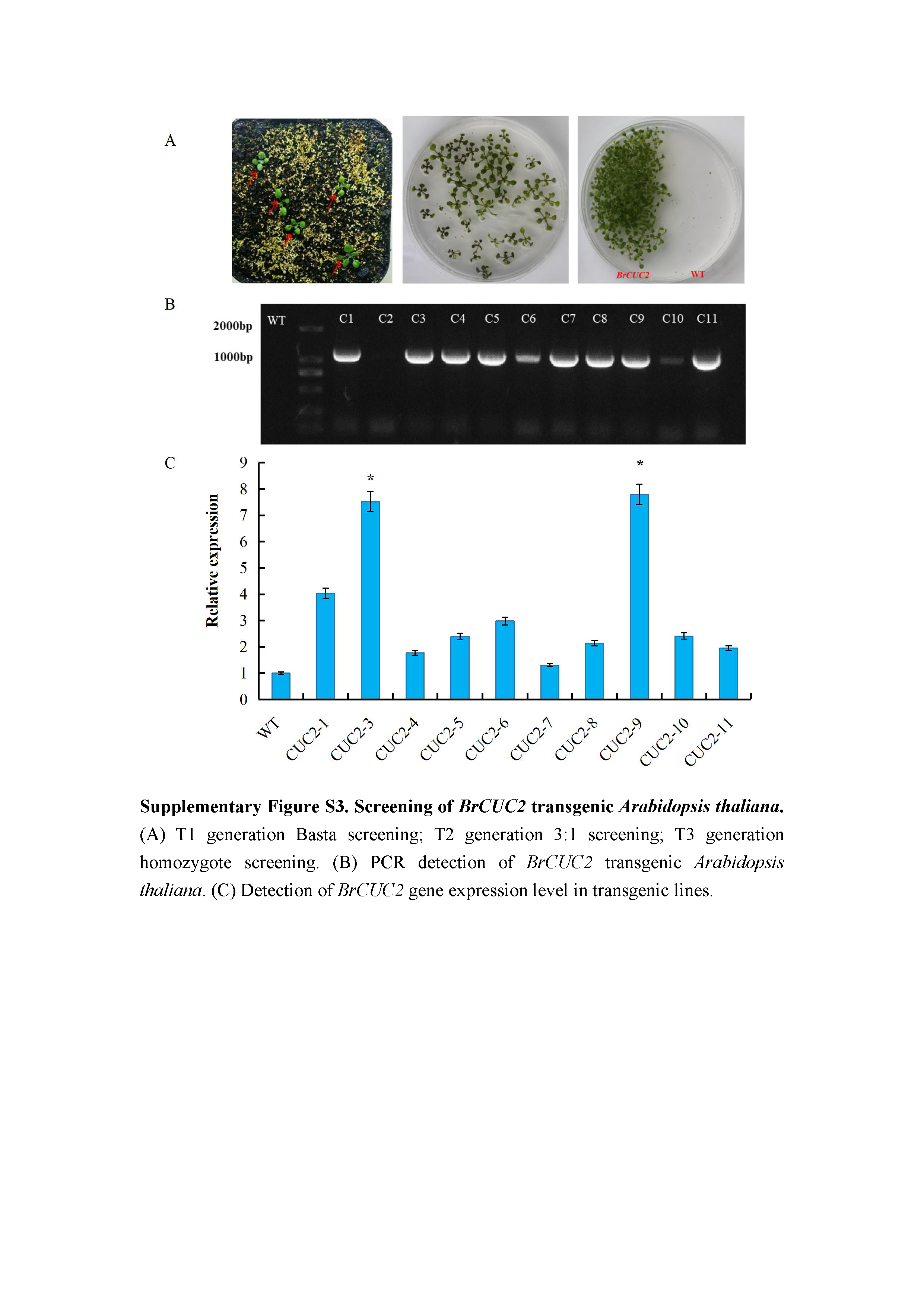

Supplement: Supplementary Figure 3 — Screening of BrCUC2 transgenic Arabidopsis thaliana. [file Image_3.tif]
